# Skin Sympathetic Nerve Activity is Modulated during Slow Sinusoidal Linear Displacements in Supine Humans

**DOI:** 10.3389/fnins.2016.00039

**Published:** 2016-02-16

**Authors:** Philip S. Bolton, Elie Hammam, Kenny Kwok, Vaughan G. Macefield

**Affiliations:** ^1^School of Biomedical Sciences and Pharmacy, University of NewcastleCallaghan, NSW, Australia; ^2^Hunter Medical Research InstituteCallaghan, NSW, Australia; ^3^School of Medicine, Western Sydney UniversitySydney, NSW, Australia; ^4^Institute for Infrastructure Engineering, Western Sydney UniversitySydney, NSW, Australia; ^5^Neuroscience Research AustraliaSydney, NSW, Australia

**Keywords:** SSNA, sympathetic, utricle, saccule, vestibulosympathetic reflexes

## Abstract

Low-frequency sinusoidal linear acceleration (0.08 Hz, ±4 mG) modulates skin sympathetic nerve activity (SSNA) in seated subjects (head vertical), suggesting that activation of the utricle in the peripheral vestibular labyrinth modulates SSNA. The aim of the current study was to determine whether SSNA is also modulated by input from the saccule. Tungsten microelectrodes were inserted into the common peroneal nerve to record oligounitary SSNA in 8 subjects laying supine on a motorized platform with the head aligned with the longitudinal axis of the body. Slow sinusoidal (0.08 Hz, 100 cycles) linear acceleration-decelerations (peak ±4 mG) were applied rostrocaudally to predominately activate the saccules, or mediolaterally to predominately activate the utricles. Cross-correlation histograms were constructed between the negative-going sympathetic spikes and the positive peaks of the sinusoidal stimuli. Sinusoidal linear acceleration along the rostrocaudal axis or mediolateral axis both resulted in sinusoidal modulation of SSNA (Median, IQR 27.0, 22–33% and 24.8, 17–39%, respectively). This suggests that both otolith organs act on sympathetic outflow to skin and muscle in a similar manner during supine displacements.

## Introduction

Interaction between the vestibular and autonomic nervous system is now well established in humans (see Yates et al., 2014, for Review). Both electrical (galvanic) and natural (head displacement) stimulation of the vestibular system have been shown to modulate sympathetic outflow to the lower limbs, influencing blood volume redistribution and thus blood pressure regulation. Galvanic vestibular stimulation (GVS), which selectively activates primary afferents arising from the peripheral vestibular organs (Minor and Goldberg, 1991; Fitzpatrick and Day, 2004), has been shown to modulate both muscle (MSNA) and skin (SSNA) sympathetic nerve activity. However, dynamic (sinusoidal) rather than static GVS is required to generate bursts of MSNA and SSNA that are phase locked to the GVS (Bolton et al., 2004; Bent et al., 2006; Grewal et al., 2009; James et al., 2010). When applied at low frequencies (0.08–0.18 Hz) that are dissimilar to the cardiac (arterial baroreceptor) frequency (~1 Hz), sinusoidal GVS (sGVS) generates two bursts of modulation of MSNA and SSNA for each stimulus cycle (Hammam et al., 2011, 2012), which reflects bilateral input from the vestibular labyrinth (El-Sayed et al., 2012).

Since GVS may influence the firing of all afferents in the vestibular nerve, whether arising from the saccule, utricle, or ampullae organs, its use does not allow determination of which components of the peripheral vestibular organ are responsible for the modulation of MSNA or SSNA (Fitzpatrick and Day, 2004). We have sought to overcome this problem by applying natural stimulation to the otolith organs in such a way as to predominantly activate hair cells in either the saccule or utricle.

Although it is known that the otolith organs are slightly curved (Curthoys et al., 1999, 2009), the majority of afferents activated by linear acceleration in the vertical plane will arise from the saccule, while acceleration in the horizontal plane will predominately activate afferents from the utricles (Fernández and Goldberg, 1976). Using this attribute we have demonstrated that slow sinusoidal horizontal accelerations-decelerations (~4 mG, 0.08 Hz) of seated (head vertical) adults, which preferentially activate the utricular organs, induce entrained modulation of MSNA when displaced in the rostrocaudal (antero-posterior) axis (32%) or medio-lateral axis (29%) (Hammam et al., 2013, 2014b). Similarly, we have shown that marked entrained modulation of SSNA occurs in seated subjects when displaced antero-posteriorly or medio-laterally, suggesting that activation of the utricle results in strong modulation of SSNA (Grewal et al., 2012). This is in contrast to Ray and colleagues, who report that while MSNA was changed by head down neck flexion from a head up posture, which arguably changes the bias of saccule hair cells activation due to gravity (Shortt and Ray, 1997), there was no change in SSNA (Ray et al., 1997). By lying subjects supine with the head aligned with the body and applying dynamic physiological stimuli involving slow linear acceleration-decelerations (peak acceleration ±4 mG) along the rostrocaudal axis to predominately stimulate the saccule we have demonstrated that MSNA modulation was similar to that when displaced mediolaterally, suggesting that both the saccular and utricular organs modulate MSNA (Hammam et al., 2014a).

In this study we examined whether preferential physiological stimulation of the *saccule* modulates SSNA. Based on our previous findings that SSNA is strongly modulated (>90%) in seated subjects displaced antero-posteriorly or mediolaterally which predominately activates the utricles (Grewal et al., 2012) and the previous report by Ray et al. (1997) that head down neck flexion in prone subjects does not modulate SSNA, we hypothesized that SSNA would be modulated in supine subjects during slow sinusoidal linear acceleration imposed medio-laterally, which predominately activates the utricle, but not during rostro-caudal accelerations that predominately activate the saccule.

## Methods

Experiments were performed on 8 subjects (5 male, 3 female; 18–58 years), following approval by the Human Research Ethics Committee at Western Sydney University, and satisfied the Declaration of Helsinki. Each participant provided written informed consent prior to commencing the experiments. All participants abstained from any caffeinated beverages for at least 3 h before the experiment. None of the participants smoked. Subjects' lay supine on a comfortable bed, neck aligned with the spine. The head was stabilized with a Velcro strap and padding to avoid rotational movements. A blindfold, earplugs and earmuffs were applied to prevent any visual or auditory cues. The bed was fixed on a motorized platform driven by two linear motors that had a maximal excursion of ±20 cm in the rostrocaudal (X) and mediolateral (Y) directions. Accelerations were measured using two high-sensitivity accelerometers, with a threshold of < 10 μG (QA650, Honeywell, USA), fixed to the platform—one orientated to record X-axis and, the other, Y-axis displacements. Sinusoidal movements of the platform (0.08 Hz and ±4 mG) were delivered separately in the X or Y directions. Each displacement had a peak-to-peak excursion of 15 cm and was performed for 100 cycles. Due to the arrangement of the hair cells within the otoliths, accelerations rostrocaudally (X-axis) cause displacement of most hair cells within the saccule, whilst mediolateral movements (Y-axis) cause displacements of most of the hair cells within the utricle, but relatively few hair cells in the saccule. At the conclusion of each stimulation sequence, subjects were asked to report their perceptions.

Skin sympathetic nerve activity (SSNA) was recorded from cutaneous fascicles of the left common peroneal nerve via a tungsten microelectrode (FHC, Bowdoinham, ME, USA) inserted percutaneously at the fibular head; an uninsulated reference microelectrode was inserted subdermally ~1 cm away. Intraneural stimulation (0.01–1.0 mA, 1 Hz, 0.2 ms pulses), delivered to the microelectrode via an isolated stimulator (Stimulus Isolator, ADInstruments, Sydney, Australia) used to guide the microelectrode tip into nerve fascicles. Neural activity was amplified (gain 20,000, bandpass 0.3–5.0 kHz; NeuroAmp EX, ADInstruments, Sydney, Australia) and the microelectrode tip manually advanced toward spontaneous bursts of oligounitary. The oligounitary activity was identified as SSNA if it had the following characteristics: (1) negative going spikes that (2) exhibited a burst in its activity when a brisk sniff was performed *and*, with the subject's eyes closed, a burst could be evoked by arousal involving an unexpected tap on the nose or loud shout (Delius et al., 1972), (3) there was no sustained increase in burst amplitude and frequency during inspiratory-capacity apnoea which is known to occur in MSNA (Macefield and Wallin, 1995). Neural activity was stored on a computer (10 kHz sampling) using a computer-based data acquisition and analysis system (PowerLab 16SP hardware and LabChart 7 software; ADInstruments, Sydney, Australia). We concurrently recorded the ECG (0.3–1.0 kHz), with Ag–AgCl surface electrodes on the chest sampled at 2 kHz, respiration measured using a piezoelectric transducer (DC-100 Hz; Pneumotrace, UFI, Morro Bay CA, USA) wrapped around the chest, and non-invasive continuous blood pressure was recorded from a finger (Finometer, Finapres Medical Systems, The Netherlands). Skin blood flow was recorded from a finger pad (photoelectric pulse plethysmograph, ADInstruments, Sydney, Australia). Baseline activity was recorded for 5min followed by acceleration of the platform, separately, in the X or Y directions at 0.08 Hz for 100 cycles (~21 min each).

SSNA was displayed as the raw neurogram as well as the RMS-processed (root mean square, moving average over 200 ms) SSNA signal. The primary analysis was conducted on the raw, negative-going, sympathetic spikes, as described previously (Bent et al., 2006). Negative-going spikes in the neurogram (with a half-width of 0.2–0.5 ms), positive-going spikes (R waves) in the ECG and the positive peaks of the accelerometer signals were detected using window discriminator software (Spike Histogram for Macintosh v2.2, ADInstruments, Sydney, Australia). As done by us previously, the same software program was used to construct auto-correlation and cross-correlation histograms (El-Sayed et al., 2012; Hammam et al., 2014a). The program identifies each defined event (SSNA spike) and plots the time of the current event (time 0) and the times of events before (negative times) and after (positive times) the current event. Consequently, the periodicity of the SSNA relative to its-self (auto-correlation) or relative to the displacement measured with the accelerometer (cross-correlation) can be illustrated in the form of a histogram. Cross-correlation histograms (50 ms bins) were constructed between SSNA and the positive peaks of the accelerometer signals in the X or Y directions (100 cycles). The histogram data was exported to a statistical and graphical analysis program (Prism 6.0 for Macintosh, GraphPad Software, USA) to fit the data to a smoothed polynomial. Lower-order polynomials were used to fit curves to the cardiac and respiratory auto-correlation histograms while higher-order polynomials were required to fit curves to the slower vestibular cross-correlation histograms. The polynomials were adjusted to create a smooth graph of the cross-correlograms. The purpose of smoothing cross-correlation histograms between sympathetic nerve activity and platform motion is to eliminate any cardiac related peaks. This enables us to further examine the nerve activity more accurately with respect to the sinusoidal platform motion. The cardiac, respiratory and vestibular (platform) modulation of SSNA was quantified by measuring the difference in the number of spikes on the smoothed curve at the peak of the modulation and at the trough. This difference was then expressed as a percentage by employing the following formula: modulation index (%) = [(peak - trough)/peak] × 100.

The data sets were tested for normality using the D'Agostino-Pearson normality test. As the data sets were non-parametric the Wilcoxon matched pairs signed rank test was used to compare differences in vestibular and cardiac modulation indices at rest and during displacements in the X and Y-axes. All statistic analysis was performed using a computer software package (Prism 6 for Mac, GraphPad Software Inc., USA). Descriptive data are reported as the median and interquartile range (IQR). The level of statistical significance was set at *p* < 0.05.

## Results

Stable oligounitary recordings of SSNA with signal to noise ratios >2:1 were obtained from 8 subjects during slow sinusoidal linear accelerations (0.08 Hz, ±4 mG) applied rostrocaudally along the longitudinal (X) axis and in 7 of the subjects when applied in the mediolateral direction (Y). One subject was unable to complete the mediolateral displacements as he became restless after completing the first 20-min recording period, in which movements were delivered in the rostrocaudal axis. Half of the subjects did not sense movement during the displacements. Three subjects sensed that they moved but were not able to identify the direction or rate of the displacement. One subject sensed movement in only one direction and correctly sensed the direction as mediolateral. No subject reported discomfort or nausea.

**Figure 1:**
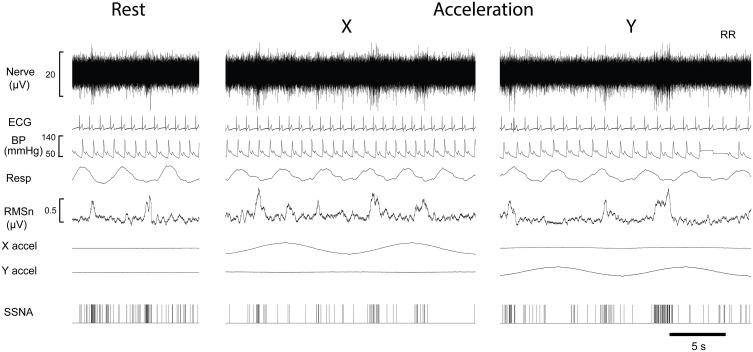
**Experimental records from a single male subject before and during sinusoidal linear acceleration in the X-direction (i.e., in the longitudinal axis of the body) and the Y- direction (i.e., mediolaterally, left to right)**. Negatively going bursts of skin sympathetic nerve activity is reflected in the RMSn processed signal. Negative going sympathetic spikes are shown discriminated below (SSNA); these were used to generate the cross-correlation histograms between the vestibular (peak acceleration) or cardiac (R-waves, ECG) signals. The acceleration (4 mG) (X accel, Y accel) caused no overt changes in heart rate, blood pressure or respiration.

Figure 1 shows the experimental records of one subject and show that there was no overt change in the blood pressure, heart rate, respiratory rate or skin blood flow during displacements. Negative-going sympathetic spikes were discriminated and are represented as spikes (SSNA). These spikes were used to generate cross-correlation histograms between SSNA and (i) the R wave of the ECG (ii) Respiration (inspiratory peak), and (iii) peak of X and Y acceleration signals.

**Figure 2:**
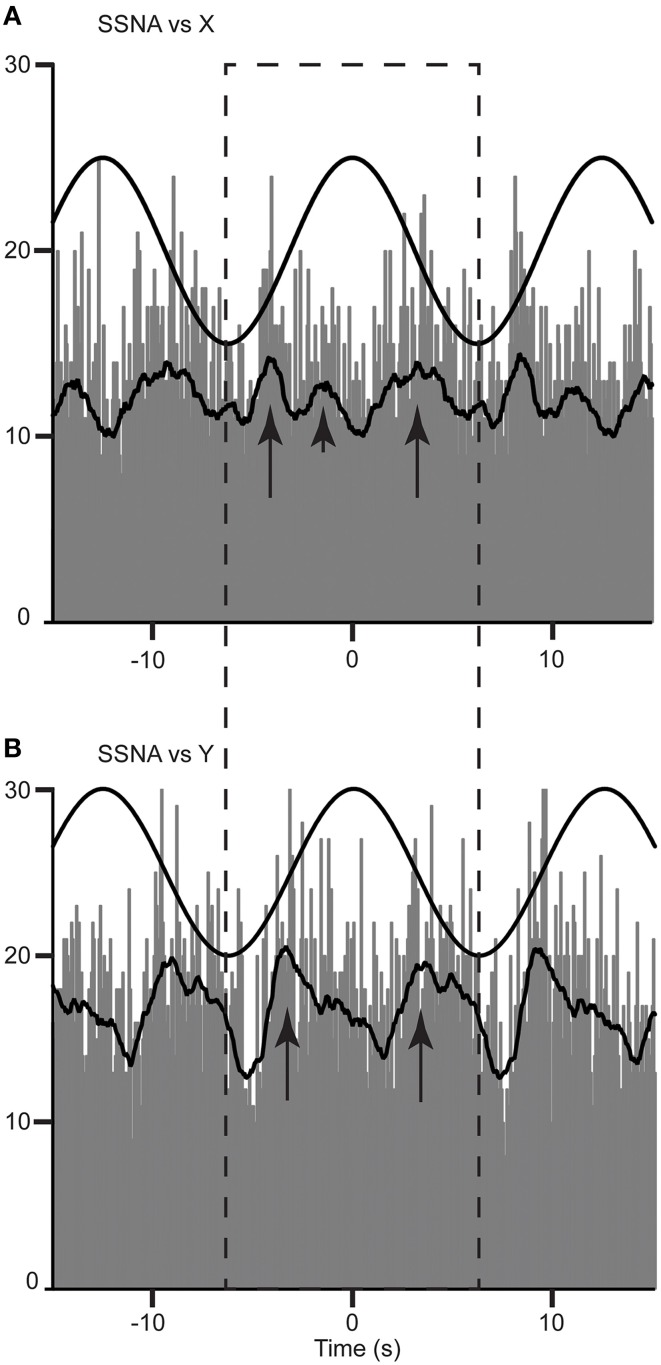
**Cross-correlation histograms between SSNA and acceleration in the rostrocaudal (X) (A) and mediolateral (Y) (B) axis in a female subject**. The histograms have been fitted with a smooth polynomial. The superimposed sinusoid represents the acceleration of the platform in a rostrocaudal **(A)** and mediolateral **(B)** axes. The long and short arrows identify the primary and secondary peaks, respectively, occurring in a single cycle defined by the dashed box.

A cross-correlation histogram of SSNA and acceleration during sinusoidal displacement in the rostrocaudal (X) and mediolateral direction (Y-axis) for one subject are shown in Figure 2. Clear modulation of SSNA occurs during each cycle and can be seen to occur as major (primary) and minor (secondary) peaks of modulation during both mediolateral (Figure 2A) and rostrocaudal (Figure 2B) displacements. Six of the eight subjects had two major peaks and two had only one major peak during each cycle of the rostrocaudal (X) displacement. Mediolateral (Y) displacement resulted in two major peaks in six subjects, and one subject had only one major peak. Most (5/7) subjects had the same number of major peaks of modulation during both rostrocaudal and mediolateral displacement. Minor or secondary peaks were present in five subjects during rostrocaudal displacement and four subjects during mediolateral displacements; three subjects exhibited secondary peaks in both rostrocaudal and mediolateral displacements.

Table 1 summarizes the vestibular and cardiac modulation of the SSNA during rostrocaual (X) and mediolateral (Y) displacements in this study. Not unexpectedly, since it is known that SSNA has only weak cardiac pulse rhythmicity (Delius et al., 1972; Macefield and Wallin, 1999), the cardiac modulation was small relative to our previous study of MSNA in supine subjects in whom we imposed the same acceleration-deceleration stimulus paradigm (Hammam et al., 2014a).

**Table 1:** Vestibular and cardiac modulation of SSNA and MSNA^*^ during sinusoidal acceleration.

**Modulation Index**	**X axis**		**Y axis**	
	**SSNA**	MSNA^*^	**SSNA**	MSNA^*^
Vestibular (%)	27.0 (22–33)	32.3 (23–38)	24.8 (17–39)	30.6 (21–40)
	[27.4 ± 4.2]	[29.4 ± 3.4]	[27.6 ± 5.0]	[32.0 ± 3.9]
Cardiac (%)	25.0 (17–33)	86.1 (83–90)	30.3 (14–37)	86.2 (81–90)
	[25.3 ± 3.5]	[86.3 ± 1.7]	[27.9 ± 4.8]	[86.2 ± 1.6]
*n*	8	12	7	10

*Modulation Index = [(peak-trough)/peak] × 100, calculated from the smoothed cross-correlation histograms (primary peak) between SSNA and the vestibular or cardiac inputs. Median (IQR) and [Mean ± SEM] data are presented. n, number of subjects*.

* *MSNA data from Hammam et al. (2014a)*.

**Figure 3:**
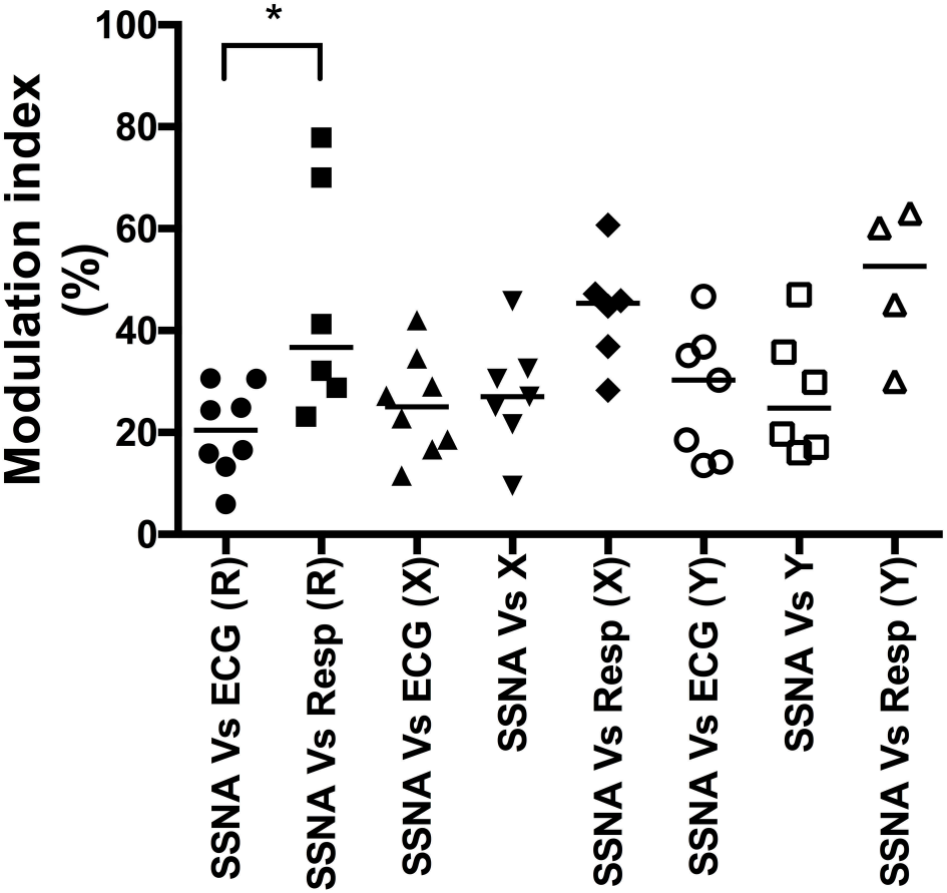
**Composite column scatter plots of the modulation indices of primary peaks of SSNA, and respective group median (horizontal bar), as a function of direction of sinusoidal motion**. There was a significant difference (^*^p < 0.05) in the magnitude of the cardiac (ECG) and respiratory (Resp) modulation index at rest (R). There was no significant difference between the respective cardiac, respiratory or vestibular modulation indices during the X or Y displacement.

The population data for the cardiac, respiratory, and major (primary) peaks of vestibular modulation are shown in Figure 3. The predominant modulator of SSNA in our supine subjects at rest was respiration, which had a modulation index that was significantly (*p* = 0.03) greater (Median, IQR; 36.7, 27–72%) than the cardiac modulation of SSNA (20.4, 14–29%). While there appeared to be an increase in the magnitude of cardiac modulation of SSNA during both X (25.0, 17–33%) and Y (30.3, 14–37%) displacements compared to the resting state, this was not statistically significant. There was also no statistically significant difference in the magnitude of the respiratory modulation of the SSNA during displacement, in either the X or Y direction, compared to that at rest and no significant difference in the respiratory modulation during the X (45.4, 35–51%) or Y (52.7, 34–62%) displacement. Similarly, there was no significant difference in the magnitude of the primary vestibular modulation of SSNA occurring during rostrocaudal and mediolateral displacement.

## Discussion

This study has demonstrated that, like MSNA (Hammam et al., 2014a), SSNA of supine humans is modulated by low-frequency, low-amplitude, sinusoidal accelerations. Furthermore, we have shown that the magnitude of the primary modulation is similar whether displaced along the rostrocaudal (27.0, 22–33%) or the mediolateral (24.8, 17–39%) axis. This provides further evidence that sympathetic outflow to the lower limbs of humans is modulated by both the saccule and utricule afferent information during displacement of the head.

We know the input from primary afferents innervating hair cells in the macula of the otolith organs is polarized, with each afferent having a direction or vector of best response to linear force sufficient to induce shear across the macula (Fernandez et al., 1972). As such, when the head is held erect (vertex to zenith) afferents from saccular hair cells will be activated during vertical displacements of the head while those from the utricle will be activated during horizontal displacements (Fernández and Goldberg, 1976).

Since the medial aspect of the saccule macula is slightly concave some hair cells will be activated with anteroposterior displacements when the head is erect. The utricular macula surface is not only concave but also inclined 30° superiorly from the horizontal at its anterior margin so some utricular hair cells will be activated by vertical displacements when the head is erect. Consequently, by placing subjects supine and thus rotating the otolith organs posteriorly by 90°, we posit that sinusoidal displacement about the horizontal plane in this study predominately activated saccular hair cells and some utricular hair cells when moving rostrocaudally (X-axis). In contrast, when moving mediolaterally (Y-axis) utricular hair cells were activated. We note, however, that—in contrast to the saccule macula—the utricle is not firmly fixed to the temporal bone and so it has been proposed that hair cells in the utricular macula could be activated as a result of the utricular macula moving under the otoconial membrane (Curthoys et al., 2009). However, hair cells do not respond to compressive forces (Goldberg et al., 2012) and so there would be little input from utricular hair cells with vectors not aligned with the primary direction of acceleration.

SSNA is known to have strong respiratory modulation (Delius et al., 1972; Fatouleh and Macefield, 2013) and this was clearly evident in our recordings. However, the respiratory modulation was not significantly different whether at rest or during displacement of our subjects. While skin sympathetic outflow involves both sudomotor and cutaneous vasoconstrictor neurons our SSNA recordings were performed in a temperature regulated environment and so were predominately associated with cutaneous vasoconstrictor neurons, which, as occurred in our study, exhibit weak cardiac rhythmicity (Delius et al., 1972; Bini et al., 1981; Macefield and Wallin, 1999; James et al., 2010).

There was clear displacement modulation of SSNA in the current study that was comparable in magnitude whether displaced in the rostrocaudal (X; 27.0, 22–33%) or the mediolateral (Y; 24.8, 17–39%) direction. The existent of two primary modulation peaks during a single cycle in either direction was consistent with the pattern of modulation previously reported following slow (0.08 Hz) sinusoidal electrical (sGVS) or natural stimulation of the peripheral vestibular system in seated (Hammam et al., 2011; Grewal et al., 2012) and supine subjects (Hammam et al., 2014a). These primary peaks of modulation likely represent shear forces acting on the hair cells in one direction and then the next when the subject is displaced rostrally and then caudally (X axis), or left and then right during mediolateral (Y) displacements (Grewal et al., 2012). While the majority of subjects also exhibited secondary peaks, we do not understand why some subjects did not exhibit this, other than that it may simply be due to a lower signal:noise ratio in these subjects.

The magnitude of the primary peaks of modulation was similar to that evoked by electrical stimulation using sGVS of seated (head erect) subjects (29.6 ± 3.0%), albeit reported as the mean ±SEM, who do not experience symptoms of nausea during sGVS (Hammam et al., 2012). However, the modulation was considerably less than that reported as the mean ±SEM for SSNA in seated subjects during anterior to posterior (96.7 ± 2.5%) or mediolateral (91.0 ± 4.7%) displacements (Grewal et al., 2012). Sauder et al. (2008) have shown that there is more sensitive and greater modulation of MSNA when in the upright posture. This is not unexpected since upright posture unloads the baroreceptors and increases baseline sympathetic activity and thus the extent to which MSNA can be modulated. However, the same mechanism cannot account for postural differences in SSNA since the baroreceptor reflex has limited modulatory effect on SSNA (Macefield and Wallin, 1999). We cannot account for the difference in SSNA modulation in the upright and supine postures, but note that the magnitude of cardiac modulation of SSNA reported in the Grewal et al. (2012) paper was comparable to that we had reported in a more recent study that compared respiratory and cardiac modulation of MSNA and SSNA (Fatouleh and Macefield, 2013).

Based on the report that, in contrast to MSNA, SSNA modulation was absent during head down neck flexion in prone subjects (Ray et al., 1997) and that slow sinusoidal physiological stimulation of the utricle induces marked entrainment (>90%) of SSNA in seated subjects (Grewal et al., 2012), we hypothesized that the utricle was the predominant vestibular modulator of SSNA. However, the current study suggests that both the saccule and utricle have comparable impact on the modulation of sympathetic outflow to the lower limb. Using the same stimulus paradigm to preferentially stimulate the saccule and utricle, we previously found that the saccule and utricle modulation of MSNA were of similar form (2 major peaks of modulation/stimulus cycle) and magnitude reported initially as the mean ±SEM (29.4 ± 3.4% and 32.0 ±3.9%, respectively) (Hammam et al., 2014a). For comparison, we now also report the magnitude of the MSNA from Hammam et al. (2014a) as the median (IQR) being 32.3 (23–38%) and 30.6 (21–40%) respectively (see also Table 1). Together these studies suggest that the saccule and utricle afferent signals make an equal contribute to vestibular modulation of sympathetic outflow to the lower limbs. Certainly it is known that ipsilateral afferents from the saccule and utricle converge on individual vestibular neurons (Kushiro et al., 2000). Studies in non-human primates have shown that vestibular nucleus neurons void of semicircular canal inputs (otolith neurons) exhibit spatiotemporal processing (Angelaki and Dickman, 2000). However, it is not clear if these neurons include those located in the caudal vestibular nuclei which are known to be involved in vestibulo-autonomic interactions, at least in quadrupeds (Miller et al., 2008) Therefore, the central effects of saccule and utricular afferents on sympathetic outflow may not be direction specific but simply facilitate up or down regulation of up-stream processing that governs sympathetic nerve outflow (Miller et al., 2008).

## Methodological limitations

We cannot exclude the possibility that some hair cells in the utricle may have contributed to the input during the rostrocaudal displacements used to stimulate hair cells in the saccule. As discussed above, any activation of hair cells in the utricle during the rostrocaudal displacements is likely to have had minimal impact given the small region of hair cells in the utricular macula that would be activated. Consequently we believed our rostrocaudal directed displacement optimized the stimulation of hair cells in the saccular macula and similarly that the mediolateral displacement primarily activated utricle hair cells.

It is also possible that since we displaced the whole body we may have activated extra-vestibular gravity sensitive (gravi) receptors that respond to blood volume and fluid shifts (Mittelstaedt, 1992). The evidence of the existence of these receptors arises from the use of lower body positive and negative pressure of the order of 30 mmHg which evokes a sense of head-down tilt and head up tilt, respectively (Vaitl et al., 2002). However, we used very small linear stimuli (±4 mG) that were below the threshold (5–6 mG) for perception of movement (Hammam et al., 2014b). As our subjects did not perceive changes in position or a sense of head tilt any blood shift induced by the displacements used in our study were unlikely to have contributed to the modulation of sympathetic outflow. Furthermore, the use of much higher linear stimulation (±260 mG) has been reported to induce increases in heart rate, and respiration that are not seen in patients with chronic bilateral vestibular dysfunction (Jáuregui-Renaud et al., 2006) suggesting that these changes require input from the vestibular system and cannot be driven by extra-vestibular gravi-receptors alone.

## Conclusions

Our study has demonstrated that slow sinusoidal linear acceleration of supine subjects induces significant modulation of SSNA that is similar in magnitude to cardiac modulation of SSNA. The modulation is of similar form and magnitude whether the displacement is along the longitudinal (X) or mediolateral (Y) axes. As we have reasoned on well-established functional anatomical grounds (Fernández and Goldberg, 1976) that our longitudinal stimulus predominately activates hair cells in the saccule and that the mediolateral displacements predominately stimulated the utricular hairs cells, we conclude that our finding that the SSNA modulation is comparable when applied along the longitudinal and the mediolateral axes together suggests that the saccule and utricle afferent information equally contribute to vestibulosympathetic reflexes. We have also observed this in a similar study of MSNA (Hammam et al., 2014a). Together, these studies strongly suggest that input from both saccule and utricle are equally involved in the modulation of vasoconstrictor drive to both muscle and skin and may thus influence blood pressure regulation in the lower limb.

## Author contributions

PB, EH, and VM conceived and designed the study. KK designed and developed the linear acceleration platform. PB, EH, and VM conducted the experiments. PB performed initial analysis and prepared draft manuscript. EH and VM reviewed data interpretation. EH, KK, and VM edited and approved final manuscript.

## Funding

This work was supported by a grant from the Australian Research Council to KK and VM (ARC DP 1096179).

### Conflict of interest statement

The authors declare that the research was conducted in the absence of any commercial or financial relationships that could be construed as a potential conflict of interest. The reviewer, VZ, and handling Editor declared their shared affiliation, and the handling Editor states that the process nevertheless met the standards of a fair and objective review.
